# CO Oxidation over Pd/ZrO_2_ Catalysts: Role of Support′s Donor Sites

**DOI:** 10.3390/molecules21101289

**Published:** 2016-09-27

**Authors:** Aleksey A. Vedyagin, Alexander M. Volodin, Roman M. Kenzhin, Vladimir V. Chesnokov, Ilya V. Mishakov

**Affiliations:** 1 Boreskov Institute of Catalysis SB RAS, Novosibirsk 630090, Russian; volodin@catalysis.ru (A.M.V.); romankenzhin@gmail.com (R.M.K.); chesn@catalysis.ru (V.V.C.); mishakov@catalysis.ru (I.V.M.); 2 Institute of Natural Resources, National Research Tomsk Polytechnic University, Tomsk 634050, Russian; 3 Institute of Power Engineering, National Research Tomsk Polytechnic University, Tomsk 634050, Russian

**Keywords:** heterogeneous catalysis, Pd catalysts, zirconia, active sites, CO oxidation, EPR, spin probe

## Abstract

A series of supported Pd/ZrO_2_ catalysts with Pd loading from 0.2 to 2 wt % was synthesized. The ZrO_2_ material prepared by a similar technique was used as a reference sample. The samples have been characterized by means of transmission electron microscopy (TEM), X-ray diffraction analysis (XRD), X-ray photoelectron spectroscopy (XPS), temperature-programmed reduction (TPR), testing reaction of ethane hydrogenolysis (HGE), N_2_ adsorption, and electron paramagnetic resonance (EPR) spectroscopy. 1,3,5-trinitrobenzene was used as a probe molecule for the EPR spin probe method. The catalytic performance of samples was tested in the model reaction of CO oxidation. It was shown that the concentration of donor sites of support measured by EPR spin probe correlates with catalytic behavior during light-off tests. Low concentration of donor sites on a support’s surface was found to be caused by the presence of the specific surface defects that are related to existence of coordinately unsaturated structures.

## 1. Introduction

The role of the atomically dispersed forms of the supported precious metals (PM) in the course of catalytic reactions is now intensively discussed in literature [1,2,3,4,5,6,7]. It seems to be rather obvious that the reduction of particle size of the supported PM (or its oxide) would cause the increase in its effective surface. At the same time, the transfer from nanosized particles to the atoms or ions stabilized on a carrier’s surface could lead to a radical change in their coordination environment that, in turn, might have a strong effect on catalytic activity. It should be noted that stabilization of a major part of the supported metal in the form of the isolated atoms or ions is caused by the existence of coordinatively unsaturated structures on an oxide surface, and can be only observed at a very low concentration of introduced PM. In the case of Pt/γ-Al_2_O_3_ catalyst, the role of such centers could be played by the unsaturated penta-coordinated Al^3+^ species, which are believed to anchor the atoms of supported metal, thus preventing them from possible agglomeration at elevated temperatures [5,6].

Similar results have been recently reported for the series of Pd/γ-Al_2_O_3_ catalysts active in the CO oxidation containing very low concentration (less than 0.5 wt %) of the supported metal [7]. On the other hand, the important role of the electron–donor surface sites in stabilization of the isolated Pd atoms in Pd/MgO system was previously shown [8]. The presence of these donor sites has been well-known for a long time to be a feature for a surface of γ-Al_2_O_3_ support. They can be tested and studied by means of specially developed spin probe techniques based on electron paramagnetic resonance (EPR) [9,10,11]. The surface concentration of such donor sites (stipulated by the presence of coordinatively unsaturated species) was found to be rather low (less than 1% of a monolayer). Now, there is no reliable data concerning the structure of such centers.

We had earlier reported about the defining role of the intrinsic donor sites of Al_2_O_3_ support in stabilization of the atomically dispersed ionic forms of supported palladium [12,13,14]. The measured concentration of such sites on alumina surface was rather low. Thus, the effect of Pd introduction had therefore become the most apparent in the case of its low concentrations (<0.5 wt %). The efficiency of using 1,3,5-trinitrobenzene (TNB) as a spin probe for studying such systems was demonstrated in the same reports [12,13,14]. Interaction of TNB molecules with the donor sites present on surface of Al_2_O_3_ support [10,15,16] and Pd/Al_2_O_3_ catalysts [12,13,14] results in emergence of stable radical anions. Identification of sites on Al_2_O_3_ surface, which are responsible for stabilization of isolated Pd^2+^ ions showing the highest activity in the CO oxidation reaction, should be mentioned as the most important result of recent studies. The EPR-detectable radical anions of TNB were established to represent an effective spin probe method applicable for investigation of active sites in Pd/Al_2_O_3_ catalysts. It was claimed [12,13,14] that the deposition of palladium on the surface of SiO_2_ and TiO_2_ oxides (known to have no intrinsic donor sites at all) does not lead to emergence of such centers in the corresponding Pd/SiO_2_ and Pd/TiO_2_ catalysts.

One may suppose that the oxide carriers having their own donor sites could behave similarly to Al_2_O_3_, thus being capable of stabilizing the supported palladium in the same way. ZrO_2_ can be named as one of the most convenient and well-studied supports for such experiments. This support was earlier studied in details and shown to possess the intrinsic donor sites [17,18,19]. On the other hand, in many cases, ZrO_2_ is used as the carrier (or as the component of the combined support) for the synthesis of PM-based catalysts [20,21].

In the present paper, we have attempted to diagnose the Pd-containing sites arising at deposition of Pd on ZrO_2_ using the EPR-based spin probe technique, for the purpose of establishing the possible correlation between the concentration of such sites and the catalytic activity of Pd/ZrO_2_ system in the CO oxidation reaction. Additionally, some of the samples were characterized by other techniques in order to prove the assumptions made.

## 2. Results and Discussion

### 2.1. Characterization of Prepared Samples

**Figure 1:**
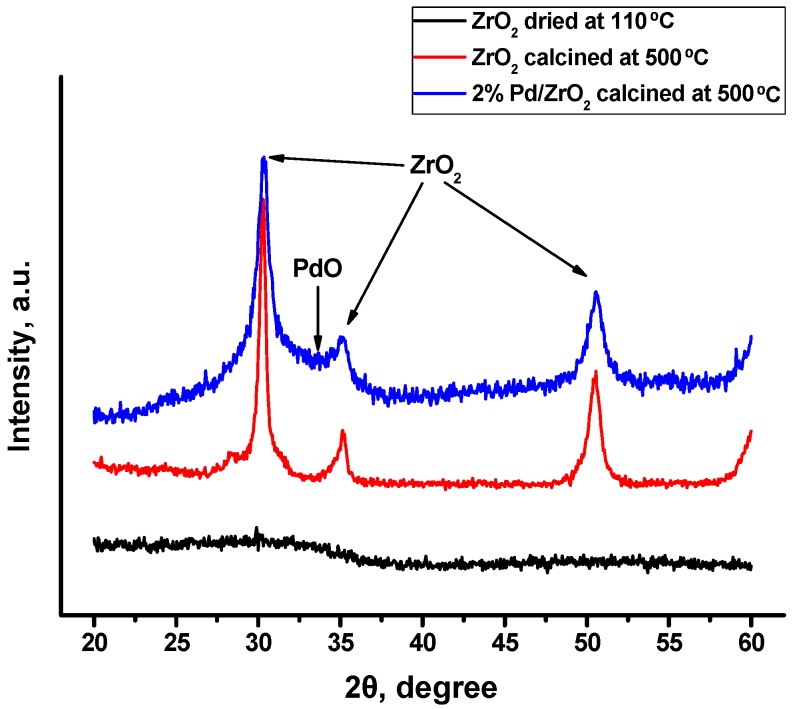
X-ray diffraction patterns for the pristine ZrO_2_ (dried at 110 °C and calcined at 500 °C) and 2% Pd/ZrO_2_ (calcined at 500 °C).

The prepared samples of pure and Pd-containing ZrO_2_ were examined by means of X-ray diffraction analysis (XRD). As it follows from Figure 1, the pristine ZrO_2_ sample dried at 110 °C is amorphous. Calcination of the sample at 500 °C leads to an appearance of reflexes assigned to crystalline ZrO_2_. The phase modification of zirconia (cubic or tetragonal) is known to be difficult to distinguish by means of XRD due to similar lattice parameters [22]. At the same time, broad peaks of ZrO_2_ phases testify to a presence of the crystallites of small size. Additionally, it should be noted that, among all Pd-containing samples, only 2% Pd/ZrO_2_ calcined at 500 °C shows an increased area at ~33°, which can be attributed to the dispersed PdO phase.

Table 1 demonstrates results of nitrogen adsorption obtained for pure and Pd-containing samples. It is evident that palladium introduction does not noticeably affect the textural properties of zirconia. At the same time, the calcination procedure results in a collapse of porous structures of amorphous ZrO_2_, due to the crystallization process that takes place at 500 °C. Values of specific surface area decrease about two times. Sulphation of the samples followed by calcination procedure decreases these values even more significantly.

**Figure 2:**
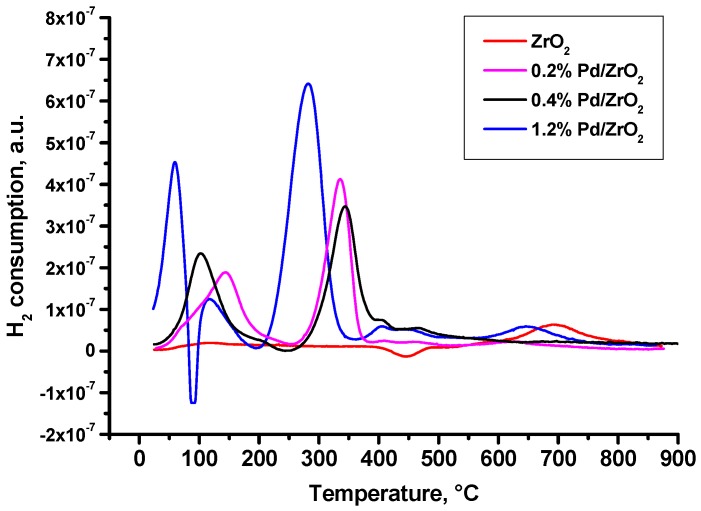
Temperature-programmed reduction profiles for the pristine ZrO_2_, and samples 0.2% Pd/ZrO_2_, 0.4% Pd/ZrO_2_ and 1.2% Pd/ZrO_2_. All samples were dried at 110 °C and calcined at 500 °C.

According to results of temperature-programmed reduction (TPR) presented in Figure 2, all Pd-containing samples contain palladium in at least in two forms: PdO particles and Pd oxide species stabilized on a surface. Increase of Pd loading changes the ratio of these forms and shifts the position of corresponding peaks. Thus, an increase of Pd content from 0.2% to 0.4% results in a slight decrease of a stabilized species and in a shift of a low temperature peak (near 100–150 °C) attributed to easily reducible forms of PdO. The appearance of a large peak assigned to bulk PdO as well as a significant shift of peak near 300 °C (indicating the weakening of metal-support interaction) was observed when the Pd loading was 1.2%. Additionally, the appearance of hydrogen evolution peak near 90 °C indicates the decomposition of PdH_2_ [11,23]. At the same time, palladium hydride is known to be formed during the reduction of bulk PdO only.

**Figure 3:**
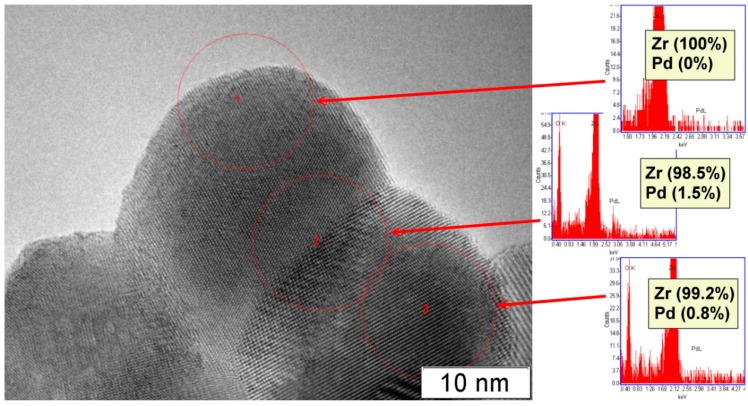
Pd concentration in different areas of 2% Pd/ZrO_2_ sample. Transmission electron microscopy and energy-dispersive X-ray spectroscopy data.

**Figure 4:**
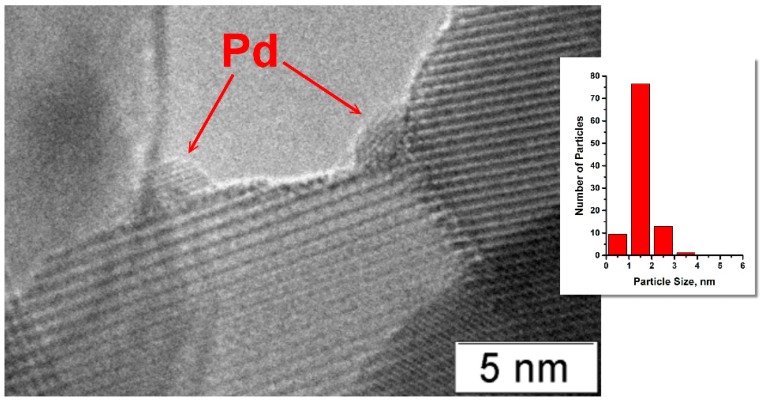
Transmission electron microscopy image of 0.8% Pd/ZrO_2_ sample after calcination at 500 °C.

Data of transmission electron microscopy (TEM) for a 2% Pd/ZrO_2_ sample shown in Figure 3 also revealed that Pd is distributed on the surface of zirconia non-uniformly. It is seen that part of Pd is located near or along the interphase boundaries of ZrO_2_. In the case of less Pd-loaded samples, the majority of the palladium species is well-dispersed and occupies these locations (Figure 4). The average particle size for 0.8% Pd/ZrO_2_ sample was 1.5 nm.

### 2.2. EPR Spectroscopy Spin Probe Method

In this work, the radical anions of 1,3,5-trinitrobenzene (TNB) were used as spin probes to characterize the active sites of Pd/ZrO_2_ catalysts. The experimental procedure was similar to that reported recently [12,13,14,15,16].

As it was shown in [12,13,14], the deposition of Pd in the presence of significant amounts of chemisorbed water significantly alters the conditions for stabilization of TNB radical anions on the donor sites of γ-Al_2_O_3_ surface. The analysis of changes in EPR spectra carried out in the works mentioned makes it possible to claim two most essential factors:

(1) Increase in average value of g-factor (g_av_) from 2.005 typical of “usual” radical anions up to 2.006 corresponding to “abnormal” ones, which can be detected only for a Pd/γ-Al_2_O_3_ system activated at low temperatures;
(2) Reduction of the constant value A_zz_ from 31 Gs for “usual” radical anions to 26.5 Gs for “abnormal” ones.

It was established that the concentration of such “abnormal‘’ radical anions correlates well with catalytic activity of samples in the CO oxidation reaction. The developed approach has been applied in present research to explore the active sites of Pd/ZrO_2_ catalysts.

**Figure 5:**
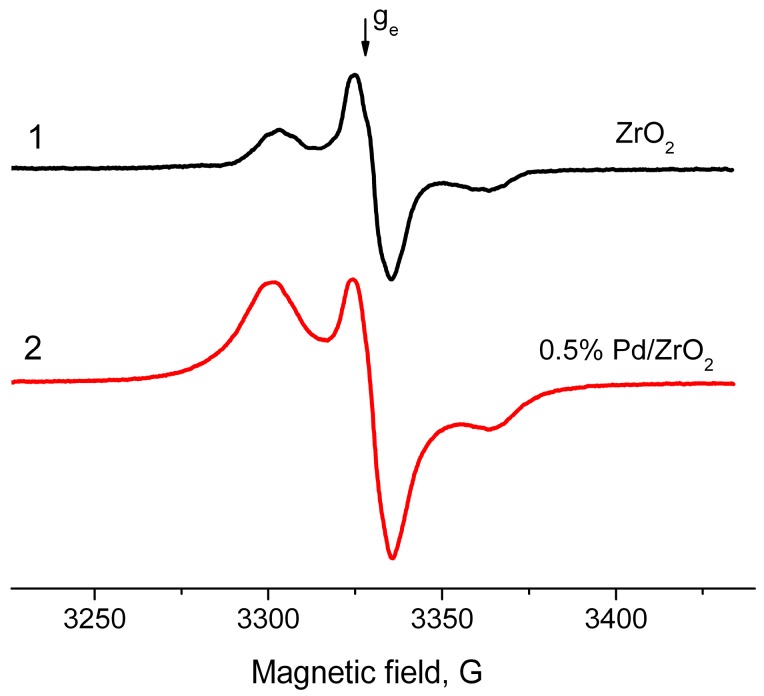
Electron paramagnetic resonance spectra of radical anions emerged after adsorption of 1,3,5-trinitrobenzene from its 2 × 10^−2^ M solution in toluene on surface of ZrO_2_ (**1**) and 0.5% Pd/ZrO_2_ (**2**) catalyst. T_act_ = 500 °C.

**Figure 6:**
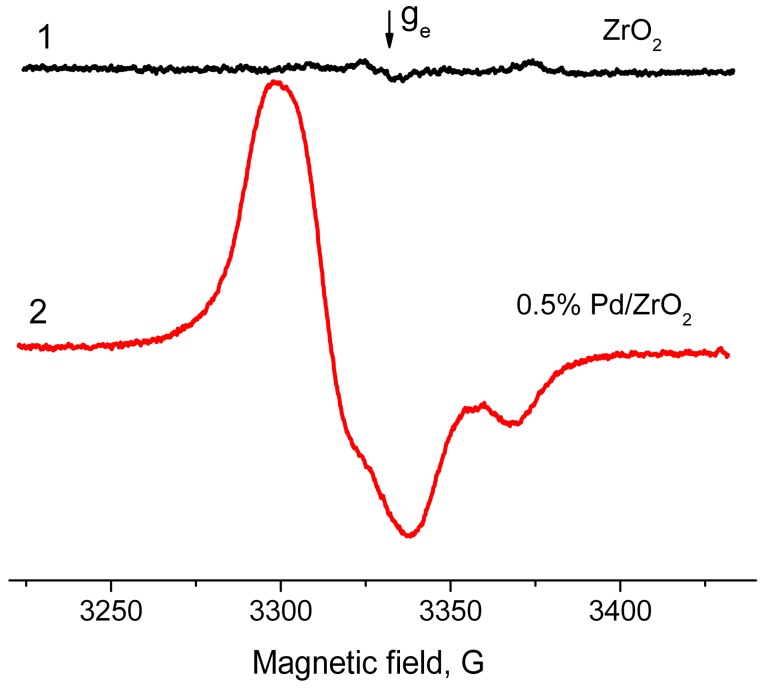
EPR spectra of radical anions emerged after adsorption of TNB from its 2 × 10^−2^ M solution in toluene on surface of ZrO_2_ (**1**) and 0.5% Pd/ZrO_2_ (**2**) catalyst. T_act_ = 100 °C.

Typical EPR spectra of the radicals arising on samples of ZrO_2_ and Pd/ZrO_2_ activated at 500 °С are given in Figure 5. In addition, in the case of Al_2_O_3_, the supported Pd/ZrO_2_ samples are characterized with much higher concentrations of radicals (2.0 × 10^18^ spin/g) as compared with ZrO_2_ (7.3 × 10^17^ spin/g). At the same time, they have the magnetic resonance parameters close to “usual” radical anions detected on surface of Al_2_O_3_ (g_av_ = 2.004, 2A_zz_ = 61 G). Meanwhile, the drastic changes were observed for the hydrated surface of ZrO_2_ and Pd/ZrO_2_ samples. Figure 6 shows the spectra of EPR radicals for these samples dehydrated at 100 °C that approximately corresponds to a monolayer of the chemisorbed water present on the surface. One can clearly see that the adsorbed water blocks the donor sites on ZrO_2_, thus suppressing the formation of radicals at adsorption of TNB (Figure 6, spectrum 1). The obtained result is found to be in good agreement with literature data concerning the high-temperature dehydration of oxide samples, which is necessary for further formation of radical anions TNB^−^. It is also worth noting that the EPR spectrum of Pd/ZrO_2_ sample exhibits rather intensive signals from radicals with g_av_ = 2.007 (Figure 6, spectrum 2).

Similar tendencies in alteration of magnetic resonance parameters are known in literature for the protonated nitroxyl radicals in solutions [24]. It is reasonable to assume that the protonation of radicals could also take place in heterogeneous system for samples of Pd/γ-Al_2_O_3_ and Pd/ZrO_2_ in the presence of significant amounts of chemisorbed water. Deposition of palladium is believed to modify the donor sites present on an oxide surface in such a way that they become less sensitive to chemisorbed water.

**Figure 7:**
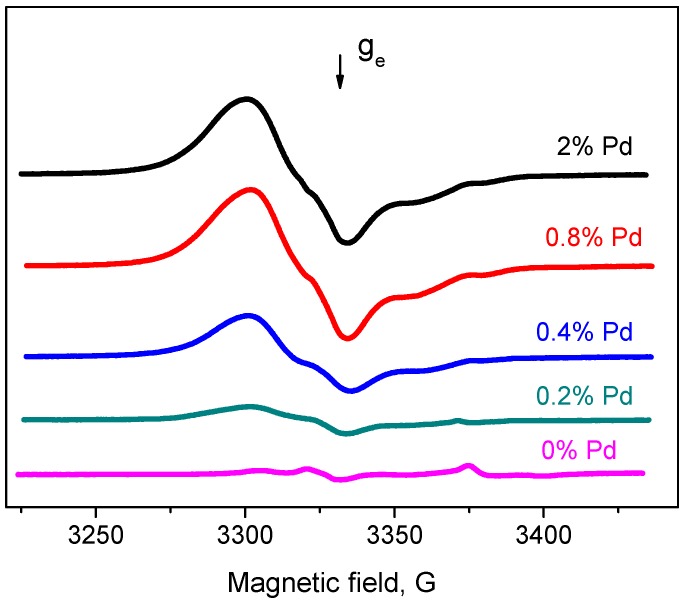
EPR spectra of TNB radical anions on the surface of x% Pd/ZrO_2_ catalyst with various concentrations of the supported Pd (x, wt %). T_act_ = 300 °C.

**Figure 8:**
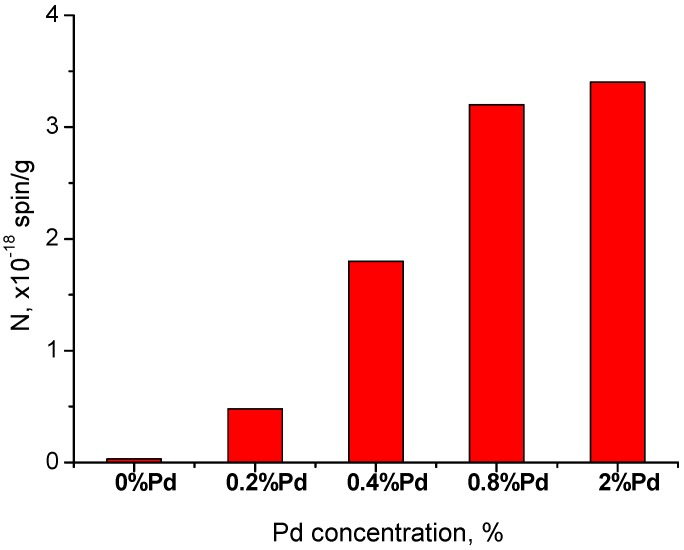
Concentration of TNB radical anions on surface of x% Pd/ZrO_2_ catalyst with various concentration of the supported Pd (x, wt %). T_act_ = 300 °C.

EPR spectra of spin probes for Pd/ZrO_2_ catalysts with various concentrations of supported Pd are given in Figure 7. Figure 8 shows the concentration of radicals corresponding to the certain x% Pd/ZrO_2_. It is well seen that the principle change in quantity of radicals is observed when the Pd loading is less than 0.8 wt %.

In general, the results obtained for the ZrO_2_ system seem to be similar to those reported earlier for Al_2_O_3_ [12,13,14]. The utmost concentration of donor sites for ZrO_2_ was measured to be ~3 × 10^18^ spin/g or ~4 × 10^16^ spin/m^2^ that corresponds to no more than 1% of a monolayer of its surface. As well as in the case of Pd/Al_2_O_3_ systems, the “abnormal” spectra of radical anion TNB^−^ (probably caused by the effect of protonation) can be observed for Pd/ZrO_2_ samples activated at low temperatures. In addition, the most expressed growth in concentrations of TNB radical anions is related to x% Pd/ZrO_2_ samples with x < 1 wt %, which is similar to observations previously conducted for the case of the Pd/Al_2_O_3_ system.

### 2.3. Testing the Catalytic Behavior in CO Oxidation Correlation with Spin Probe Data

Due to a developed EPR-based spin probe method, an atomically dispersed ionic form of Pd supported on Al_2_O_3_ has recently been found to possess extremely high catalytic activity in the CO oxidation reaction [12,13,14]. Establishment of correlation between EPR spin probe data and catalytic activity of x% Pd/ZrO_2_ catalysts is discussed in the present section.

**Figure 9:**
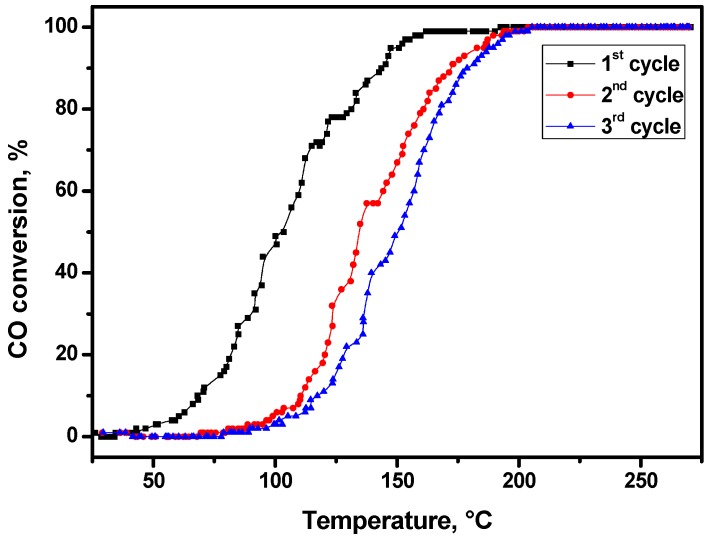
Light-off curves for the CO oxidation over 2% Pd/ZrO_2_ catalyst in three consecutive catalytic cycles.

The Pd-based catalysts used for the CO oxidation in aftertreatment systems of automobile exhausts are known to have a problem of low-temperature deactivation, which could take place during the first 2–3 catalytic cycles. The characteristic behavior of 2% Pd/ZrO_2_ catalyst within catalytic cycles can be given as an example (Figure 9). It is seen that light-off curves shift from cycle to cycle towards high temperatures, thus indicating catalyst’s deactivation. Table 2 summarizes the results of catalytic tests for all samples of studied series. As follows from the data presented, the problem of cycle-to-cycle deactivation is peculiar to Pd/ZrO_2_ catalysts, regardless of the Pd loading.

**Figure 10:**
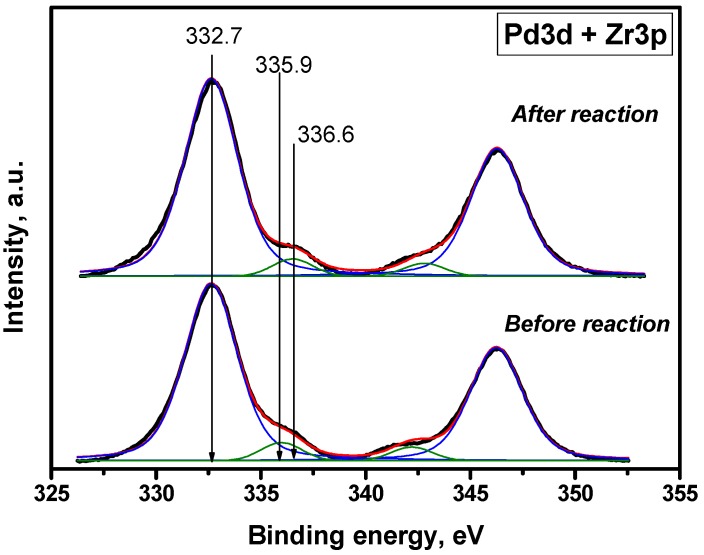
X-ray photoelectron spectroscopy spectra (Zr3p and Pd3d) of 2% Pd/ZrO_2_ (before and after catalytic cycles).

The sample with the highest Pd loading (2 wt %) before and after catalytic cycles was studied by means of X-ray photoelectron spectroscopy. Figure 10 shows the corresponding X-ray photoelectron spectroscopy spectra of Zr3p and Pd3d regions. Analysis of Pd3d region in spectra for initial catalyst (E_b_ = 335.9 eV) has shown that the palladium exists in the form of metal particles. Treatment of the sample with the reaction mixture results in an increase of E_b_ corresponding to the Pd3d region (E_b_ = 336.6 eV), which may suggest the presence of Pd in a state that is close to Pd^2+^; in particular, this value corresponds to PdO. The atomic ratio for Pd with other elements calculated from XPS data are listed in Table 3. As follows from the table, the concentration of Pd on the surface is decreased in case of sample after 3 catalytic cycles.

A similar result can be observed from the data presented in Table 4. In this case, we have examined samples with low and medium content of palladium. The numbers show the specific surface area (SSA) of Pd estimated by testing the reaction of ethane hydrogenolysis (HGE). It is evident that the specific surface area of Pd is sharply decreased from cycle to cycle. This observation can be explained by possible formation of surface carbonate-carboxylate structures, which block the active sites of the catalysts. If we compare the values of SSA for samples with different Pd loading, it is seen that initially 0.4% Pd/ZrO_2_ and 1.2% Pd/ZrO_2_ are very close to each other by this parameter. While the kinetics of the deactivation process seem to be similar and independent from Pd loading, the SSA value for low-loaded samples decreases more rapidly from cycle to cycle.

**Figure 11:**
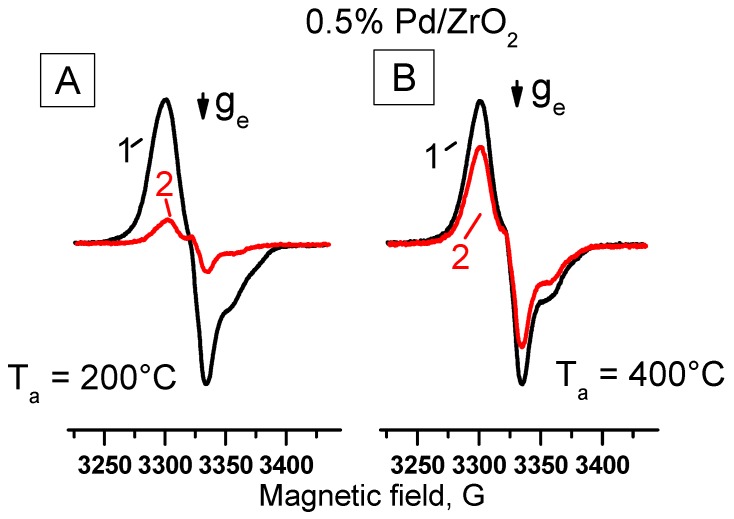
EPR spectra of radical anions resulted from adsorption of TNB on 0.5%Pd/ZrO_2_ catalyst before (1) and after three catalytic cycles (2). Prior to carrying out the experiments with spin probes, samples were heated in air at a temperature of: 200 °C (**A**); 400 °C (**B**).

It was quite reasonable to explore the state of active sites of the catalysts before and after three catalytic cycles by means of a developed spin probe technique. Figure 11 shows EPR spectra recorded for 0.5% Pd/ZrO_2_ heated in air before spin probe testing at T = 200 °C (spectrum A) and T = 400 °C (spectrum B).

As follows from Figure 11, at a dehydration temperature of 400 °С, the values of concentrations of the detected radical anions for the initial sample and the catalyst spent in three catalytic cycles practically coincide. This could be explained by the regeneration of the initial state of catalyst after such treatment. However, the reduction of activation temperature down to 200 °С (Figure 11A) results in a significant difference in TNB^−^ concentration detected for 0.5% Pd/ZrO_2_ sample before and after catalytic reaction, which correlates well with the decrease of its catalytic activity observed in the third catalytic cycle (Figure 9). Based on the results obtained, it becomes possible to claim that the donor centers in both Pd/Al_2_O_3_ and Pd/ZrO_2_ catalysts detected by the spin probe technique have a direct relationship to catalytic sites responsible for the CO oxidation. It seems to be obvious that the low activation temperature of samples when using a spin probe technique allows one to diagnose the active sites for the state, which is characteristic for the catalyst after catalytic reaction.

**Figure 12:**
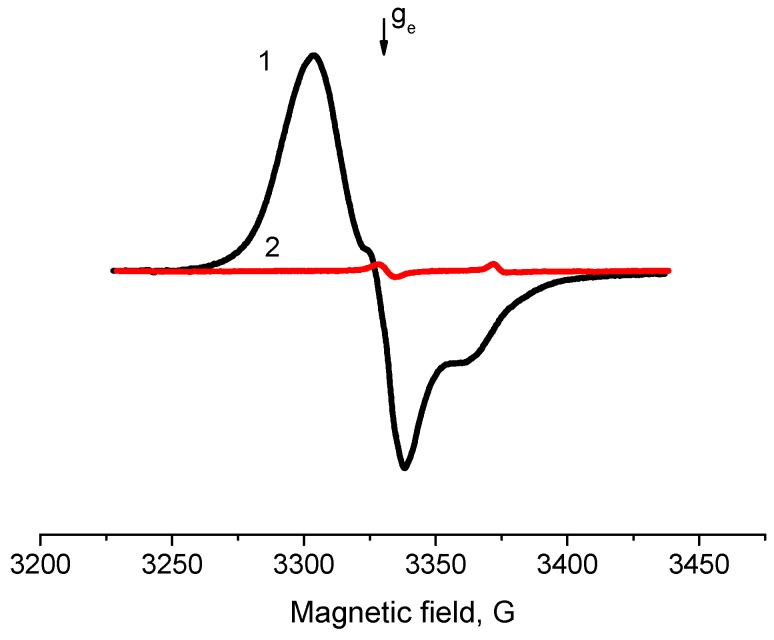
EPR spectra of TNB radical anions adsorbed on 2% Pd/ZrO_2_ (**1**) and sulfated 2% Pd/ZrO_2_ catalyst (**2**). T_act_ = 500 °C.

**Figure 13:**
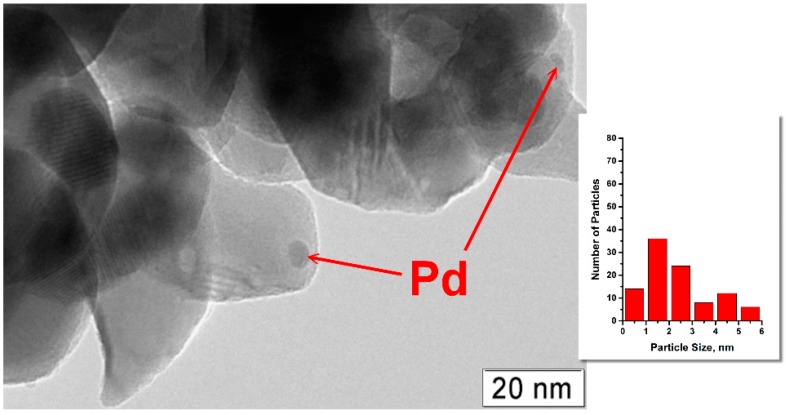
TEM image of 0.8% Pd/ZrO_2_ sample after sulphation and calcination at 500 °C.

In addition, the specially performed experiments showed that a deposition of palladium on supports characterized with the absence of donor sites (SiO_2_, TiO_2_) does not lead to emergence of the latter. At the same time, the suppression of donor sites in the Pd/Al_2_O_3_ catalyst by the sulphation, for example, leads to considerable reduction of concentration of the observed TNB radical anions. This effect is brightly illustrated in Figure 12 for the sample of 2% Pd/ZrO_2_ catalyst. Disappearance of donor sites changes the surface properties of zirconia, and whittles the stabilization effect towards palladium even at moderate Pd loading (Figure 13). In the case of 0.8% Pd/Al_2_O_3_ sample, average particle size increases up to 2.3 nm, while particle size distribution becomes wider.

**Figure 14:**
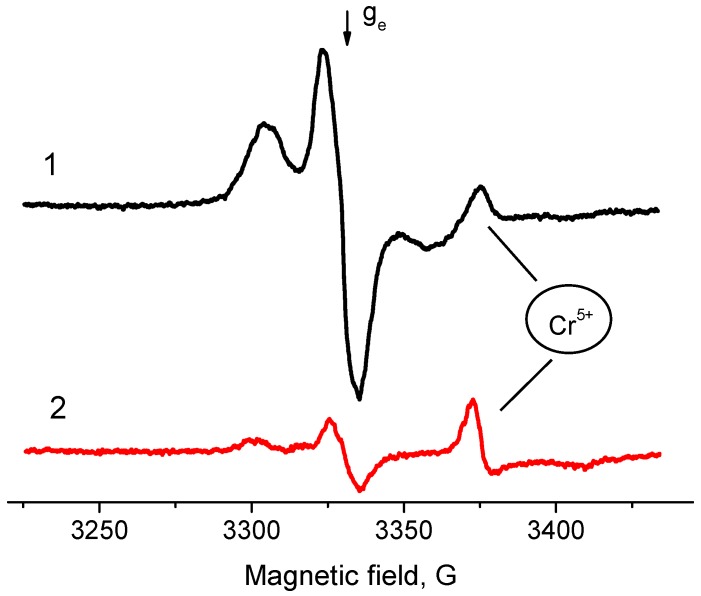
EPR spectra of TNB radical anions adsorbed on ZrO_2_ (**1**) and sulfated ZrO_2_ (**2**). T_act_ = 500 °C.

In the case of sulphation of pristine ZrO_2_ material (free from supported Pd), the reduction of donor sites concentration in comparison with starting ZrO_2_ can be observed as well (Figure 14). As it was already mentioned, the process of sulphation is accompanied with an intensive collapse of zirconia structure, which aggravates the negative effect. Presence of extrinsic Cr^5+^ ions in zirconia with corresponding signals at g_1_ ~ 1.96 and g_2,3_ ~ 1.98 is well-described in literature [18,25]. Since the concentration of these ions is negligibly low, the signal appears only in the case of samples when aimed signals from spin probes are insignificant.

For further study, it was natural to determine whether the tested species are related to the sites accounting for the catalytic activity of Pd/ZrO_2_ catalysts in CO oxidation reaction.

**Figure 15:**
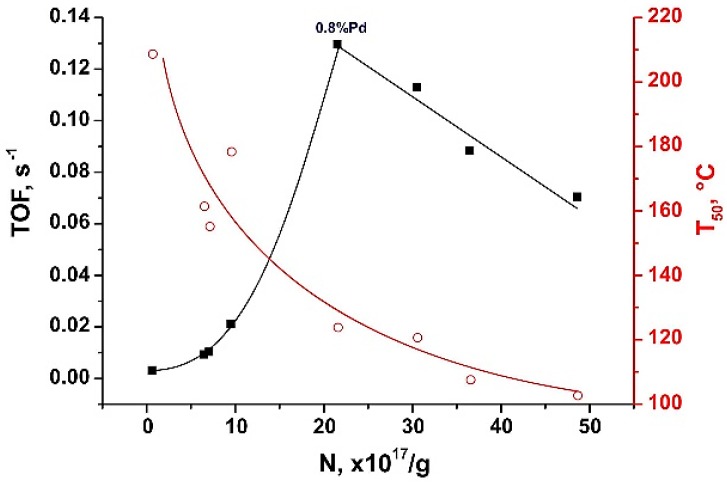
Catalytic performance of Pd/ZrO_2_ samples in the CO oxidation (turnover frequency and T_50_) vs. concentration of TNB^−^ radicals detected on their surface. TOF was calculated for temperature of 150 °C.

We used an arbitrary selection of Pd/ZrO_2_ samples for direct comparison of the concentration of donor sites (N) with their catalytic activity (turnover frequency and T_50_). The results are presented in Figure 15. One can see that there is a smooth correlation between the concentration of donor sites and T_50_ (red line). This result indicates that the donor sites tested in Pd/ZrO_2_ catalysts using the above spin probe method are involved in the studied reaction. At the same time, the dependence of turnover frequency (TOF, number of molecules of CO converted per surface atom of palladium particles and per second) on the concentration of donor sites (N) is more complicated. It can be divided into two ranges. TOF increases exponentially along with growth of N until the value corresponded to Pd loading of 0.8 wt %. Further increase of N (higher Pd loading) results in an almost linear descent of TOF. It can be concluded here that 0.8 wt % is the maximum amount of Pd, which can be evenly distributed and stabilized on the surface of ZrO_2_, providing both high concentrations of donor sites and superior catalytic activity.

## 3. Experimental Section

### 3.1. Preparation of the Samples

Samples of hydrated zirconium oxide were prepared by the conventional way based on precipitation of ZrOCl_2_ precursor with ammonia at pH = 10.6. The precipitate was washed with a great excess of water until the pH is close to 7. The synthesized samples were dried at 110 °C for 12 h and were not supposed to be exposed to any other heat treatment.

For the preparation of series of x% Pd/ZrO_2_ catalysts (x = 0.2%/2% wt %), the calculated amount of 10% aqueous solution of Pd(NO_3_)_2_ was added to the as-prepared wet precipitate of the hydrated zirconia. The obtained material was dried at 120 °C for 2 h, and then calcined at 350 °C (1 h) and further at 500 °C (1 h). The described treatment procedure is known to favor the formation of crystalline ZrO_2_ in cubic modification. The Pd-free ZrO_2_ sample was synthesized by the similar procedure to serve as a reference sample.

Sulphation of ZrO_2_ and Pd/ZrO_2_ samples was carried out by the impregnation in aqueous solution of (NH_4_)_2_SO_4_ with the subsequent drying and calcination at 500 °C. The concentration of the supported ammonium sulfate was about 4 wt %.

### 3.2. Characterization of the Samples

Specific surface area (S_BET_) of the samples was determined by low-temperature nitrogen adsorption/desorption using an ASAP 2400 instrument (Micromeritics Instrument Corp., Norcross, GA, USA).

Transmission electron microscopy images were obtained using a JEOL JEM-2010 electron microscope (JEOL, Kyoto, Japan). with lattice resolution 0.14 nm at accelerating voltage 200 kV. The local energy-dispersion X-ray (EDX) microanalysis was carried out using EDAX spectrometer (Oxford Instruments, Oxford, UK). with energy resolution 127 eV.

The phase composition of the samples was studied in 2θ range 10°–75° on a Shimadzu XRD-7000 diffractometer (Shimadzu Corporation, Kyoto, Japan) (CuKα radiation, Ni filter on the reflected beam, and a scintillation detector with amplitude discrimination). Interpretation of the diffraction patterns was done in accordance with JCPDS-ICDD (Joint Committee on Powder Diffraction Standards–International Center for Diffraction Data) database [26].

The X-ray photoelectron spectra (XPS) were obtained with a VG ESCA-3 (Vacuum Generators Scientific, East Grinstead, UK) electron with Mg Kα radiation. The binding energy of adventitious C1s (284.5 eV) was used as an internal reference. The pressure in the analysis chamber was maintained lower than 1 × 10^−7^ mbar. Energy regions were selected after a general survey and scanned with several sweeps until a good signal-to-noise ratio was observed. The accuracy of the binding energy (BE) values was ±0.1 eV.

Investigation of the samples (100 mg, fraction of 0.25–0.5 mm) by means of temperature-programmed reduction (TPR) was carried out in a flow reactor system equipped with a thermal conductivity detector (Chromatec Instruments, Yoshkar-Ola, Russia) [12]. Prior to the testing, all samples were treated in oxygen atmosphere at 400 °C for 0.5 h followed by cooling down to −10 °C. Then, the reactor was flushed with Ar flow, and the testing procedure was started. The flow rate of reduction mixture (10% H_2_ in Ar) was 40 mL/min. All samples were heated in the temperature range from 0 to 900 °C.

### 3.3. EPR Spectroscopy with Spin Probes

Electron paramagnetic resonance (EPR) spectroscopy was performed using an ERS-221 EPR spectrometer (Center of Scientific Instruments Engineering, Leipzig, German Democratic Republic) operating in the X-band (ν = 9.3 GHz). EPR spectra were acquired at 20 dB attenuation with typical microwave power 3 mW. The frequency of the microwave irradiation and the magnetic field were measured using a CZ3-64 frequency meter (Meridian, Kiev, Ukraine) and NMR magnetometer MJ-110R (Radiopan, Wroclaw, Poland), respectively. The EPR-CAD software package (Version EPR-CAD 2.1, BIC, Novosibirsk, Russia) was used to control the spectrometer operation and analyze the results obtained.

In this work, the radical anions of 1,3,5-trinitrobenzene (TNB) were used as spin probes to characterize the active sites of Pd/ZrO_2_ catalysts. A quartz sample tube with 40 mg of sample was placed in a muffle furnace and heated at 100–500 °C for 12 h. Then, the sample was cooled down to room temperature and inundated with 2 × 10^−2^ M solution of TNB in toluene. In these conditions, the formation of TNB radical anions started. To accelerate the process and achieve the maximum concentration of radical anions, which corresponds to the concentration of surface donor sites of the sample, the tube was additionally kept at 80 °C for 2 h. Then, the EPR spectra of the formed radical anions were registered at room temperature. The concentrations of the paramagnetic species were determined by numerical double integration with baseline compensation. Rigid sample placement in the spectrometer resonator and computer processing of the spectra allowed us to decrease typical errors in the determination of the absolute radical concentrations to ±10%. The average g-tensor value (g_av_) was determined by numerical integration including correction of the zero line.

### 3.4. Measurement of Palladium Surface Atoms

The amount of palladium surface atoms was determined using ethane hydrogenolysis (HGE) instead of commonly used CO chemisorption. The activity of the catalysts in the reaction of ethane hydrogenolysis is known to correlate with the specific surface area of palladium in the metallic state [27]. The initial reaction rate and the light-off temperature are usually used as criteria of the catalytic activity. Pd catalysts are active in this reaction in a temperature range of 300–500 °C.

Fresh catalyst (0.25–0.5 mm fraction, 100 mg loading) was reduced in H_2_ flow at 500 °C. Then, the reactor was cooled in the hydrogen flow to the reaction temperature. Afterwards, the helium flow was mixed with hydrogen, and the H_2_/He mixture was passed through the reactor for some time until the system reached a steady state. At this point, ethane was added to the flow. The ethane flow was passed for 3 min. Then, a probe was taken for chromatographic analysis, and the hydrocarbon flow was stopped. For 10 min of the chromatographic analysis, the sample was subjected to the H_2_/He mixture flow only. This was necessary to regenerate the initial state of the catalyst. This procedure was repeated for five times at each temperature in the studied temperature range with a step of 25 °C.

### 3.5. Testing the Catalytic Behavior in CO Oxidation

The catalytic performance of samples in a model reaction of CO oxidation was studied using a laboratory-scale light-off testing procedure. After grinding and sieving to reach the grain size in the range of 0.25 to 0.5 mm, catalyst samples were employed for testing. The feed stream consisted of 0.15 vol % CO, 14 vol % O_2_, 0.01 vol % NO, 5 vol % H_2_O and N_2_ as the balance. The total gas flow was 0.3 dm^3^/min corresponding to a feed gas space velocity (GHSV) of 180,000 h^−1^. The temperature of the catalyst was increased from 60 to 320 °C with a heating rate of 10 °C/min. The gas concentrations were determined using an Fourier transform infrared gas analyzer (Siemens, Munich, Germany). Each sample was tested in several heating-cooling cycles.

The TOF, the number of molecules of CO converted per surface atom of palladium particles and per second, was calculated for all samples. The amount of palladium surface atoms was estimated using the HGE method described above.

## 4. Conclusions

The results obtained in the present research allow one to confirm the earlier stated hypothesis concerning the important role of donor sites in stabilization of the atomically dispersed Pd species over the surface of oxide supports. The EPR-based spin probe technique previously developed for Pd/Al_2_O_3_ system turned to be an effective tool for exploration of the active sites in Pd/ZrO_2_ catalysts. The concentrations of such sites in Pd/ZrO_2_ catalysts were found to be determined by the amount of donor sites present on the surface of starting ZrO_2_ material. The defined concentration of donor sites does not exceed the size of (3/5) × 10^18^ sites/g. As soon as all donor sites are filled up with Pd clusters, the process of further Pd deposition results in formation of Pd nanoparticles, and, eventually, the PdO phase. This process was shown to not be accompanied with a noticeable increase of catalytic activity of certain samples in the CO oxidation.

The developed approach makes it possible to estimate the efficiency of various oxide carriers in terms of possible stabilization of the atomically dispersed ionic forms of Pd on their surface. Moreover, the preliminary results showed that it turns to be very effective for the testing of active sites in the mixed oxide catalysts based on Al, Zr and La oxides. In certain cases, it is possible to detect the active sites with other supported metals (Rh, Pt).

It is rather obvious that low concentration of donor sites on a carrier’s surface is caused by the presence of the specific surface defects that are related to the existence of coordinately unsaturated structures. Development of approaches to manage the number of such species on a surface of the oxide support could give an opportunity to increase the concentration of the active sites responsible for high catalytic activity of Pd-containing catalysts.

## Figures and Tables

**Table 1:** Specific surface area of pristine ZrO_2_, 0.2% Pd/ZrO_2_ and 2% Pd/ZrO_2_.

Sample	Dried at 110 °C	Calcined at 500 °C	Sulphated and Calcined at 500 °C
ZrO_2_	273	126	83
0.2% Pd/ZrO_2_	275	124	83
2% Pd/ZrO_2_	268	123	80

**Table 2:** Temperature of 50% CO conversion for series of Pd/ZrO_2_ catalysts in three consecutive catalytic cycles.

Sample	T_50_, °C		
	1st Cycle	2nd Cycle	3rd Cycle
0.2% Pd/ZrO_2_	208.6	211.7	212.9
0.4% Pd/ZrO_2_	161.3	169.1	173.5
0.5% Pd/ZrO_2_	155.1	168.3	172.6
0.8% Pd/ZrO_2_	178.3	180.7	185.1
1.2% Pd/ZrO_2_	119.0	144.1	162.3
2.0% Pd/ZrO_2_	102.6	134.1	150.4

**Table 3:** Atomic ratio of elements in 2%Pd/ZrO_2_ catalyst identified by X-ray photoelectron spectroscopy.

Sample	Pd/Zr	Pd/C	Pd/O
Before reaction	0.026	0.016	0.012
After 3 catalytic cycles	0.022	0.017	0.008

**Table 4:** Effect of number of catalytic cycles on specific surface area of Pd.

Number of Catalytic Cycles	Specific Surface Area of Palladium, m^2^/g	
	0.4% Pd/ZrO_2_	1.2% Pd/ZrO_2_
0	420	425
1	189	254
3	27	112
6	3	54
